# Aortic Dissection Presenting as Leg Pain

**DOI:** 10.7759/cureus.8389

**Published:** 2020-06-01

**Authors:** Nathan George, Latha Ganti, Michael Falgiani, Bobby Desai

**Affiliations:** 1 Emergency Medicine, Ocala Regional Medical Center, University of Central Florida College of Medicine, Ocala, USA; 2 Emergency Medicine, Envision Physician Services, Nashville, USA; 3 Emergency Medicine, University of Central Florida College of Medicine/Hospital Corporation of America Graduate Medical Education Consortium of Greater Orlando, Orlando, USA; 4 Emergency Medical Services, Polk County Fire Rescue, Bartow, USA

**Keywords:** aortic dissection, common iliac artery occlusion, thromboembolism, ct angiogram

## Abstract

A female in her mid-sixties presented to the emergency department with the chief complaint of leg pain. After evaluation, she was found to have a rapidly progressing aortic dissection resulting in an occlusion of the right common iliac artery. The authors highlight the variable presentations of acute aortic dissection, beyond classic tearing chest pain.

## Introduction

Aortic dissection is a surgical emergency with an incidence of three per 100,000 and a mortality rate of 25% to 30% [1-4]. Sudden onset tearing chest pain is the classic presentation, but aortic dissection can have a variable presentation due to the aorta’s anatomic course.

The patient was brought to the emergency department (ED) via emergency medical services (EMS). Paramedics reported that they were originally called for chest and abdominal pain, but noted that the patient complained only of severe right leg pain upon their arrival at the patient’s house. In this paper, the authors report a case of aortic dissection, outlining some of the typical features and reinforcing the point that a thorough history is paramount in not missing this life-threatening diagnosis.

## Case presentation

History of present illness

A 65-year-old female with a history of active leukemia being treated with imatinib presented to the ED via EMS with a chief complaint of right leg pain. She had no history of hypertension or connective tissue disease. Paramedics reported that they were originally called for chest and abdominal pain, but noted that the patient complained only of severe right leg pain upon their arrival at the patient’s house.

Per conversation with the patient's family, the pain was reported to have started in the patient's chest, migrated to her abdomen, and then migrated to her right leg. The patient denied having any current symptoms of the prior chest or abdominal pain. The pain started suddenly, approximately 20 minutes prior to contacting EMS for transport to the emergency department and was rated at a 10/10 severity. Upon arrival, the patient appeared to be in distress and frequently requested pain medication. The history of present illness and review of systems was limited, as the patient was distressed due to her leg pain.

The patient’s family reported that she was active and had no symptoms prior to the onset of the current problem. They indicated that she was outside mowing lawns on a riding mower for most of the day and was feeling fine at the end of her workday.

Physical examination

The patient's vital signs were as follows: temperature: 97.4^0^F, pulse 98 beats per minute (bpm), respiratory rate 20 breaths per minute, blood pressure 110/70 mmHg, and oxygen saturation 96%.

On physical examination, the patient was in moderate distress due to pain. No diaphoresis was noted. The patient was alert and oriented times three. Her head was atraumatic and normocephalic, moist mucous membranes were noted, and no lymphadenopathy was present. Her lungs were clear to auscultation bilaterally with no rales, wheezing, or rhonchi. On cardiac examination, regular rhythm with tachycardia is noted. There were no murmurs or bruits.

Pulses were noted at +2/4 in the upper extremities bilaterally and in the left lower extremity. Right lower extremity pulses were +0/4. The abdomen was soft and nontender to palpation, there was no abdominal bruit or pulsatile mass noted. There were no obvious signs of injury or deformity to the patient’s upper lower extremities. She had full strength noted in the upper extremities bilaterally in the left lower extremity. Diminished (+0/5) strength was noted in the right lower extremity at the levels below the right hip. The skin was warm and dry with no cyanosis noted. 

Neurological examination was normal with the exception for inability to move the right lower extremity or fuel stimuli below the level of the right hip. Vascular examination demonstrated absent dorsalis pedis and posterior tibial pulses in the right lower extremity upon palpation and Doppler examination. The patient denied sensation in the right lower extremity and stated that she could not move anything below her right thigh. Straight leg and range of motion testing of the affected right lower extremity showed full range of motion without pain or crepitus. The patient reported that she felt a burning sensation in her right posterior thigh with passive flexion of the thigh. Lower extremities were equal in size and temperature. There was no edema, swelling, or appearance of cyanosis in either of the lower extremities.

Diagnostic focus and assessment

**Figure 1:**
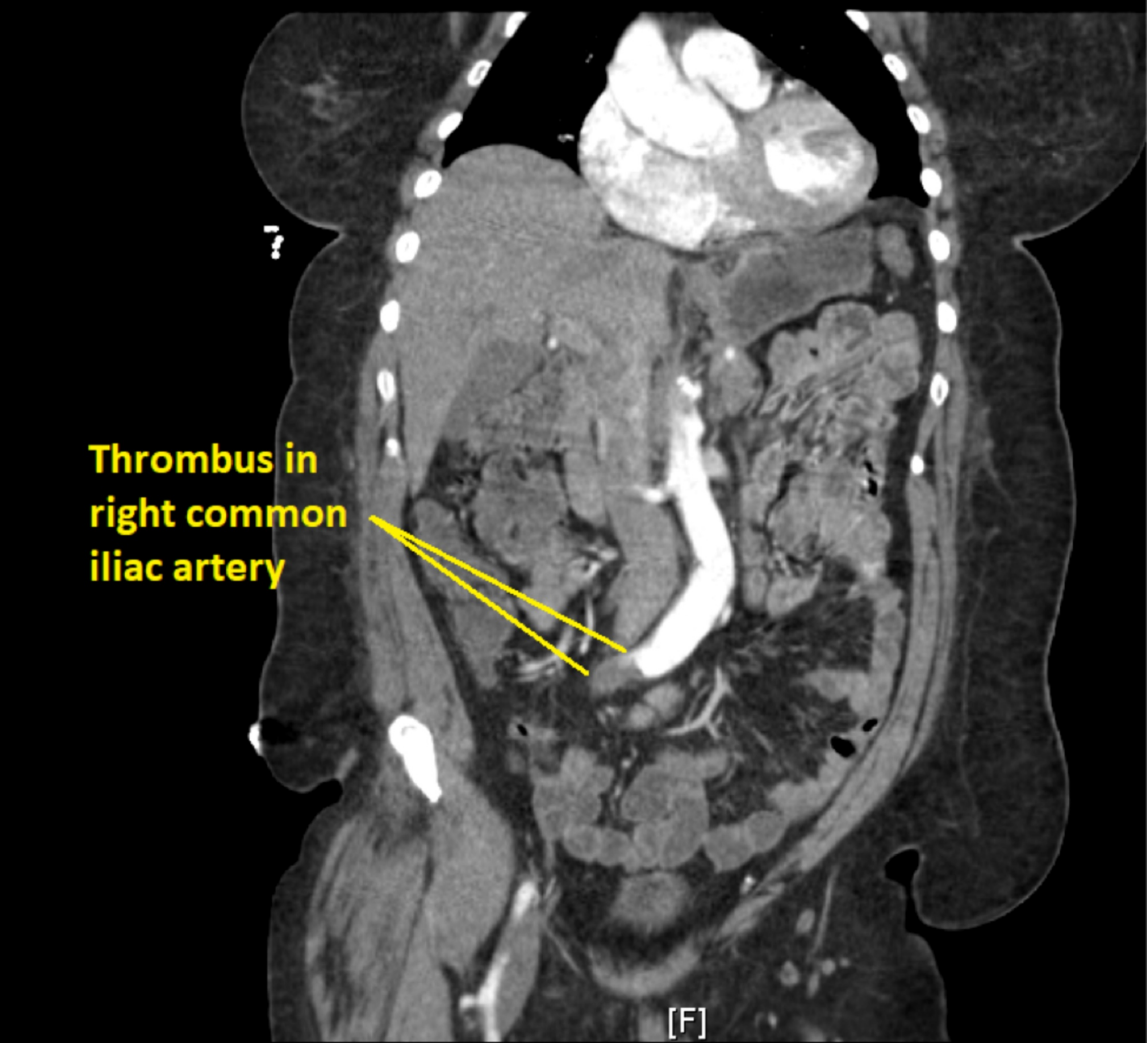
CT scan illustrating right common iliac artery occluded by a thrombus.

Based on the patient’s initial presentation of severe pain of the right lower extremity, coupled with absent pulses at the posterior tibial and dorsalis pedis pulse points, the patient was initially thought to have a high level arterial occlusion in the proximal right lower extremity. Ultrasound imaging of the lower extremity was negative for deep venous thrombosis. CT angiography of the right lower extremity was significant for thrombus occlusion of the right common iliac artery (Figure 1).

**Figure 2:**
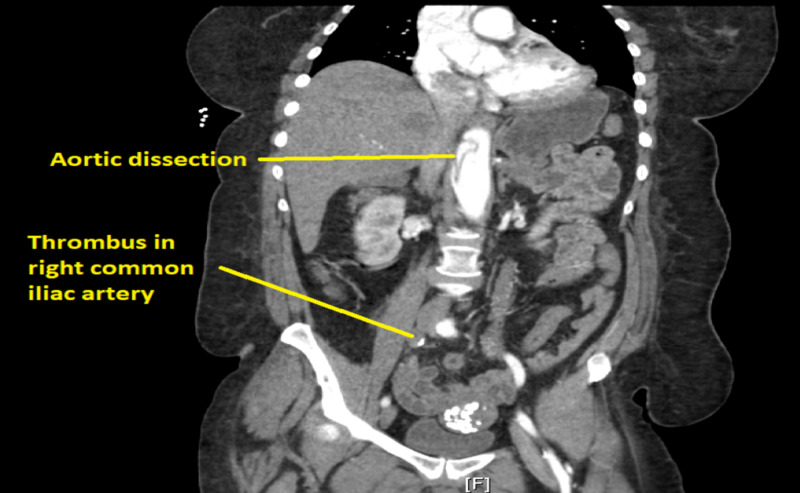
CT scan showing thrombus occluding right common iliac artery and aortic dissection.

**Figure 3:**
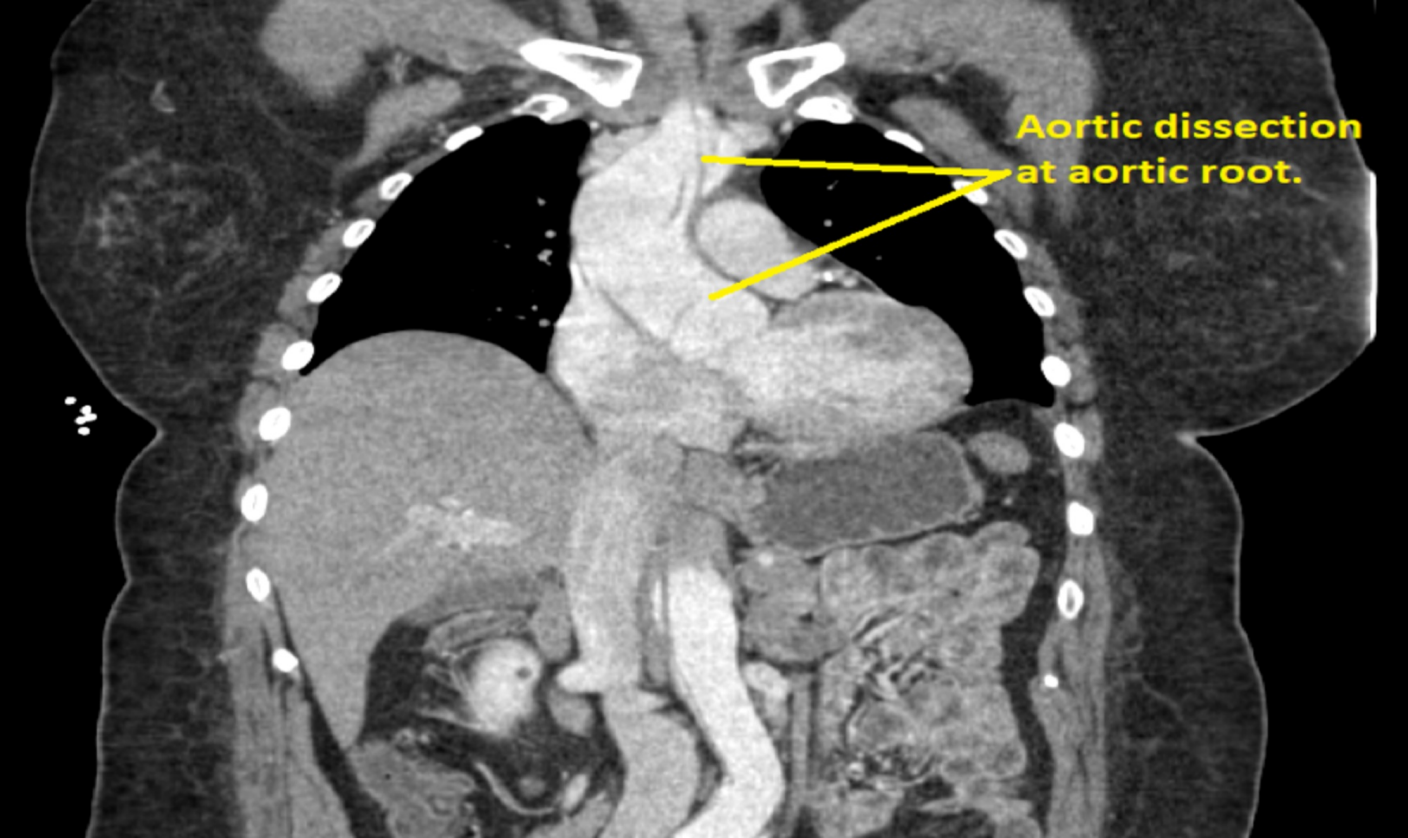
CT scan showing dissection at aortic root.

Additionally, imaging of the patient’s abdomen that was captured as part of the runout of the angiogram testing discovered an aortic dissection in the patient’s abdomen. Follow-up angiography of the patient’s chest and abdomen revealed an aortic dissection which extended from the aortic root to approximately the level of the renal arteries (Figures 2, 3).

Therapeutic focus and assessment

Based on the initial presentation of the patient (in pain), 0.5 mg of hydromorphone was administered for analgesia with another 0.5 mg administered after initial dosage, but this did not fully relieve the patient’s pain. This initial dose of pain medication alleviated the patient’s pain temporarily with enough pain relief provided to tolerate CT and ultrasound testing. Another 1 mg of hydromorphone was administered for the patient’s pain approximately 90 minutes after the initial dose for further pain control. Upon discovery of the patient’s aortic dissection and right lower extremity thrombus, esmolol and nicardipine drips were initiated with the goal of controlling the patient’s blood pressure to a level below 140 mmHg systolic and heart rate with a target of 60 bpm. Cardiothoracic surgery was contacted regarding the patient and indicated that she would require a higher level of care than the facility could provide. The patient was subsequently transferred to a facility with a higher level of care where the aortic dissection was repaired and the patient recovered well.

## Discussion

Our patient presented with leg pain, a complaint that could easily have been triaged as nonurgent resulting in delay of her ultimate high stakes diagnosis. Fortunately, she was evaluated promptly with a good history and physical examination. Nonetheless, initial evaluation centered on the leg itself, and the aortic dissection was discovered during CT angiography of the patient’s right lower extremity which prompted follow-up CT scanning of the chest to confirm the aortic dissection.

This case underscores the importance of obtaining history from both EMS and the family. It was the family who conveyed that the patient indeed had chest and abdominal pain prior to the leg pain. The patient’s initial chest pain likely signified the start of the aortic dissection. As the aorta continued to dissect, the pain migrated from the patient’s chest to the abdomen over the course of approximately 20 minutes. The resolution of the chest and abdominal pain likely signified the conclusion of the acute dissection event. Subsequent onset of right leg pain following the chest and abdominal pain is likely due to a thromboembolism or flap of aortic dissection tissue occluding the right common iliac artery. This resulted in the nonstandard chief complaint for the condition that was the root cause of the problem

One modality that may have expedited the diagnosis is ultrasonography. Abdominal aortic ultrasound, transthoracic echocardiography, and contrast-enhanced ultrasound are considered comparable to CT angiogram in detecting aortic aneurysm and aortic dissection [5].

## Conclusions

The important lesson learned in this case is that proper evaluation and physical examination of the patient is always paramount regardless of initial presentation by other providers in the healthcare system. In this case, the assessment was initially focused on the leg; although the complete diagnosis was made, it may have been made earlier had the complaint not have anchored on extremity pain.

## References

[1] Craen A, Rosario J, Amico K, Tak M, Ganti L (2019). Transthoracic echocardiographic findings of Stanford type A aortic dissection: a case report. Cureus.

[2] Melvinsdottir IH, Lund SH, Agnarsson BA, Sigvaldason K, Gudbjartsson T, Geirsson A (2016). The incidence and mortality of acute thoracic aortic dissection: results from a whole nation study. Eur J Cardiothorac Surg.

[3] Clouse WD, Hallett JW Jr, Schaff HV (2004). Acute aortic dissection: population-based incidence compared with degenerative aortic aneurysm rupture. Mayo Clinic Proc.

[4] Hagan PG, Nienaber CA, Isselbacher EM (2000). The international registry of acute aortic dissection (IRAD): new insights into an old disease. JAMA.

[5] Tsung AH, Nickels LC, DePortu G, Flach EF, Stead LG (2013). Aortic dissection and thrombosis diagnosed by emergency ultrasound in a patient with leg pain and paralysis. Case Rep Vasc Med.

